# Supplementary figures and images for: Vaccination Against Amyloidogenic Aggregates in Pancreatic Islets Prevents Development of Type 2 Diabetes Mellitus
Source: Vaccines (Basel). 2020 Mar 2;8(1):116. doi: 10.3390/vaccines8010116 (PMC7157615; doi:10.3390/vaccines8010116)

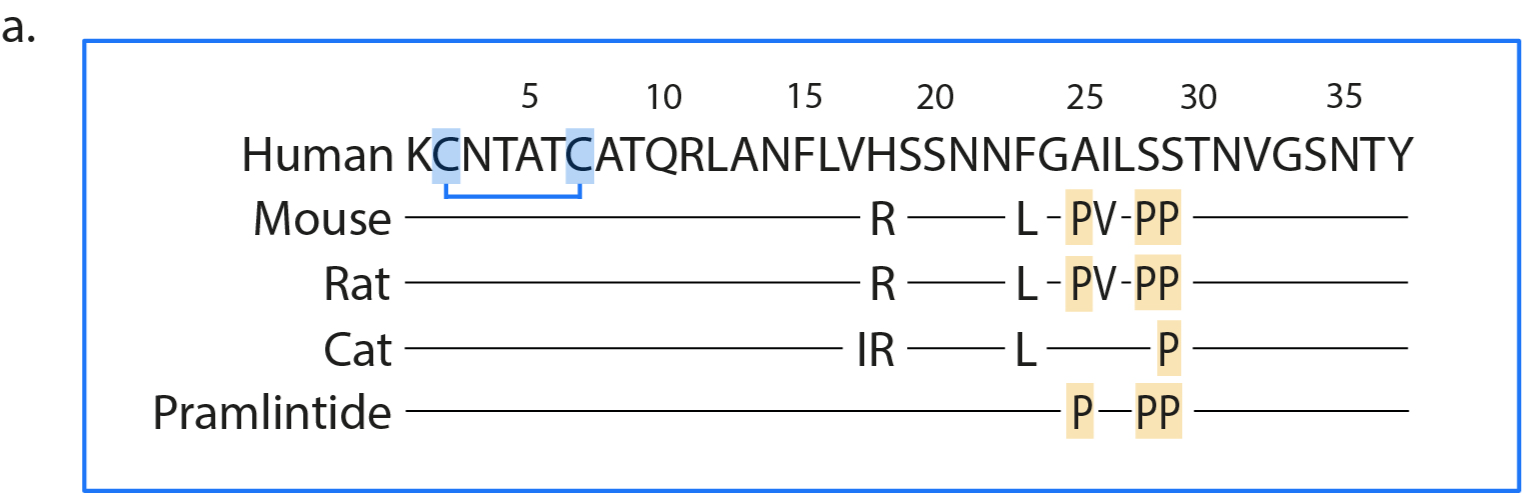

Supplement: Supplementary file 1 [file vaccines-08-00116-s001.zip › suppl.figure 1 - amylin aa sequence.tif]

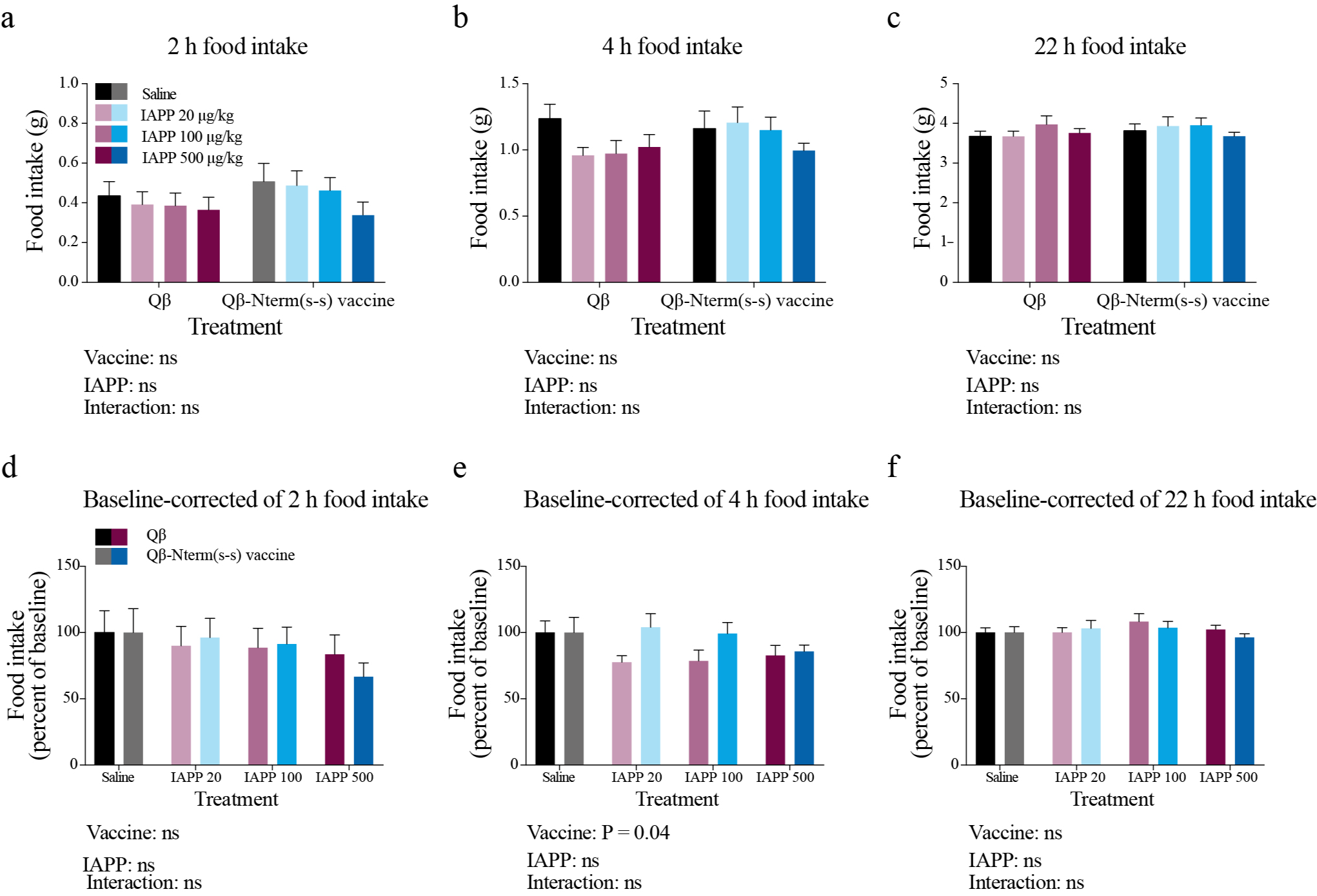

Supplement: Supplementary file 1 [file vaccines-08-00116-s001.zip › suppl.figure 2 - food intake.tif]
